# Shared Mechanisms of Neurodegeneration in Alzheimer's Disease and Parkinson's Disease

**DOI:** 10.1155/2014/648740

**Published:** 2014-05-12

**Authors:** Anmu Xie, Jing Gao, Lin Xu, Dongmei Meng

**Affiliations:** ^1^Department of Neurology, Affiliated Hospital of Medical College, Qingdao University, Qingdao, Shandong 266003, China; ^2^Department of Endocrinology, Affiliated Hospital of Medical College, Qingdao University, No. 16 Jiangsu Road, Qingdao, Shandong 266003, China

## Abstract

Alzheimer's disease (AD) and Parkinson's disease (PD) have markedly different clinical and pathological features, but these two diseases are the most common neurodegenerative disorders. Previous studies have showed that there are common mechanisms in AD and PD. Several genetic studies have revealed mutations in genes associated with the risk of AD and PD. Circumstantial evidences have shown that dysregulation of brain iron homeostasis leads to abnormal iron accumulation and results in AD as well as PD. **α**-Synuclein and tau take part in the mechanisms of these diseases by oxidative stress and mitochondrial dysfunction. Some studies indicated that the loss of LC noradrenergic neurons may occur early in the progression of AD and PD. Nicotinic acetylcholine receptors (nAChRs) are members of the Cys-loop superfamily of pentameric ligand-gated ion channels; some evidence showed that nicotinic receptors may be associated with AD and PD. These experimental and clinical studies may provide a scientific foundation for common shared mechanisms in AD and PD.

## 1. Introduction

**Figure 1:**
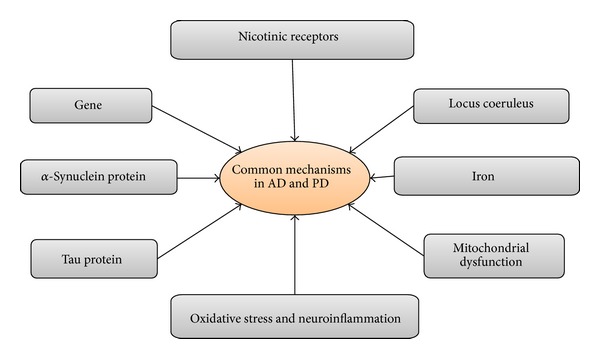
Shared mechanisms in AD and PD.

Alzheimer's disease (AD) and Parkinson's disease (PD) are the most common neurodegenerative diseases. Both of them have a large population of suffers. However, there is no cure for multifactorial diseases like AD and PD currently. Classic pathological features of AD are aggregates of insoluble amyloid beta-protein (Ab) and neurofibrillary tangles (NFTs) consisting of precipitates or aggregates of hyperphosphorylated tau protein [1]. One of the most important clinical clues to probable AD is a history of insidious learning and memory difficulties, often noticed by others and sufficient to impact performance of day-to-day activities [2]. Other deficits across a range of other cognitive faculties include higher visual processing, frontal executive function, and language abilities [3]. Pathologically, PD is highlighted by degeneration of dopamine neurons in the substantia nigra pars compacta (SNpc) as well as Lewy body (LB) or Lewy neurite (LN) intracellular inclusions largely composed of *α*-synuclein [4] (Table 1). PD, traditionally, has been defined by its characteristic motor hallmarks of rest tremor, bradykinesia, rigidity, and gait impairment [5]. Although AD and PD have distinct mechanisms of etiology, different brain regions, and distinct clinical features, they have much overlap in the development of neurodegeneration. This review aims to elucidate the common shared mechanisms in AD and PD (Figure 1) from gene mechanisms to histological level. Next, we will share these mechanisms.

## 2. Gene

Despite AD and PD being clinically distinct entities, there is a possible pathological overlap. AD and PD have been hypothesized to share common genetic determinants. Lots of studies have suggested that some genes are associated with AD as well as PD.

Some genome-wide association (GWA) studies have suggested that the two diseases represent a biological continuum. The application of GWA studies to idiopathic forms of AD and PD has identified a number of loci containing genetic variants that increase the risk of these two disorders. Previous studies performed a combined PD-AD meta-analysis and compared the results with those obtained in the primary GWA studies. The findings imply that loci increase the risk of both PD and AD and that the pathological overlap could instead be “downstream” of the primary susceptibility genes that increase the risk of each disease [6]. The role of PON1 in AD and PD is important because of its putative biological roles in pesticide metabolism, inflammation, and oxidative stress as well as the involvement of these mechanisms in the pathogenesis of neurodegenerative disease. Case-control studies of PON1 genetic variants in AD and PD have revealed positive albeit inconsistent association with two PON1-coding polymorphisms: Q192R (rs662) and L55 M (rs854560). Positive studies showing an association between PON1 polymorphisms rs662 and rs854560 in AD and PD may be partially explained by failure to adjust for relevant covariates [7, 8] or by population stratification as evidenced by departure or near departure from HWE [9]. The death of individuals with QQ192 at a younger age could cause erroneous conclusion that RR192 or RQ192 is associated with AD or PD [10]. Glutathione S-transferase omega-1 and glutathione S-transferase omega-2 genes (GSTO1 and GSTO2), residing within an AD and PD linkage region, have diverse functions including mitigation of oxidative stress and may underlie the pathophysiology of both diseases. GSTO polymorphisms were also reported to associate with risk and age at onset of these two diseases. These findings reported SNPs, GSTO1 rs4925 and GSTO2 rs156697, in AD and PD in association with disease risk, age at diagnosis, and brain gene expression level [11]. Otherwise, a variant in the NEDD9 gene may be another common genetic factor in AD and PD. Chapuis et al. data indicated that the SNP rs760678 of NEDD9 gene is at best a weak genetic determinant of AD or PD [12].

In conclusion, these studies of PON1, GSTO, and NEDD9 genes showed that AD and PD shared common genetic mechanisms.

## 3. ***α***-Synuclein

*α*-Synuclein (*α*-SN) is a ubiquitous 140-amino acid protein of 18–20 kDa that is encoded by a single gene consisting of seven exons borne by chromosome 4 [13]. Some studies indicated that *α*-synuclein had association with AD and PD.

*α*-SN is implicated in the pathogenesis of AD. *α*-SN has been suggested to be involved in aberrant synapse formation in the brain of AD patients [13]. Increased intensity of *α*-SN staining has been found in the brain of AD patients compared with controls [13]. In early stage of AD, accumulated *α*-SN can be detected at the presynaptic site suggesting that such abnormal deposit may be an early event in the pathogenesis of AD [14]. *α*-SN is reported to be found in the plaques in human AD brain as well as Tg2576 AD model mice brain, suggesting that full-length *α*-SN is released by neurons, either as part of normal cellular processing or, alternatively, as a result of neuronal degeneration and death [15, 16]. *α*-SN is the precursor protein of a nonamyloid *β* component of senile plaques (NACP) in Alzheimer's disease (AD) [13]. Lewy bodies and Lewy neurites are frequently observed in AD [17]. About 40–50% of AD patients have *α*-SN positive Lewy bodies [18]. Recent reports from the Alzheimer's Disease Neuroimaging Initiative (ADNI) found increased CSF *α*-SN in patients with mild cognitive impairment (MCI) and AD [19]. Thus, *α*-SN may take part in the mechanism of AD.

A key pathological feature of PD is Lewy bodies, of which the major protein component is just *α*-SN. Human genetic studies have shown that mutations (A53T, A30P, and E46 K) and multiplication of the *α*-SN gene are linked to familial PD. Mice overexpressing the human A53T mutant *α*-SN gene develop severe movement disorders [20]. Lasnsbury et al. [21] have ascribed the pathological properties of PD mutant *α*-SN (A30P and A53T) to enhanced formation of so-called protofibrils [22]. These pathological reports implied that *α*-SN also contributes to the pathophysiology of PD.

## 4. Tau Protein

As widely known, AD is characterized at the histological level by the so-called neurodegenerative plaques and neurofibrillary tangles (NFTs) [23]. The tau proteins are microtubule-associated phosphoprotein whose levels are regulated by tau kinase and phosphatases [24]. Tau homeostasis plays an important role in the maintenance of microtubule stability, dynamics, and neuronal viability [25]. Hyperphosphorylation of tau has been verified to lead to dynamic instability and disintegration of microtubular networks and eventually to formation of NFTs [26], resulting then in neurodegeneration [25]. And the core of its constituent filaments is made of a truncated fragment from the repeat domain of tau. This truncated tau can catalyze the conversion of normal soluble tau into aggregated oligomeric and fibrillar tau which, in turn, can spread to neighboring neurons. Its initiating substrate complex is most likely formed as a consequence of a progressive loss of endosomal-lysosomal processing of neuronal proteins, particularly of membrane proteins from mitochondria [27]. Meanwhile, the abnormal phosphorylation of tau results in the formation of NFTs which is produced by the action of tau kinases, leading to the loss of neurons and synapse and eventually to dementia [24]. Thus, tau may take part in the mechanism of AD.

In addition to AD, tau also takes part in PD. Herbert et al. determined the diagnostic value of cerebrospinal fluid (CSF) DJ-1 and tau proteins for discriminating PD and multiple system atrophy (MSA). DJ-1 and total tau levels were quantified in the CSF of 43 PD patients, 23 MSA patients, and 30 nonneurological controls matched for age and gender. The result showed that the combination of DJ-1 and tau proteins significantly improved this discrimination to 82% sensitivity and 81% specificity to identify MSA from PD. The result highlighted the potential benefits of a combination of DJ-1 and total tau as biomarkers for differential diagnosis of MSA and PD [28]. Another study observed a significant correlation between CSF levels of tau proteins and *α*-synuclein in a cohort of entirely untreated patients with PD at the earliest stage of the disease. These results found a significant correlation of the levels of *α*-synuclein with the levels of T-tau and P-tau181 and found that measures of CSF A*β*1-42, T-tau, P-tau181, and *α*-SN have prognostic and diagnostic potential in early-stage PD [22]. So, tau also takes part in the mechanism of PD.

## 5. Iron

It was found that some metals, such as iron, copper, zinc, and aluminum, suffer progressive changes along the advance of neurodegeneration, suggesting that these imbalances could be related to the decline of cognitive functions.

Low molecular mass fractions of iron, copper, aluminum, and cobalt appear to play a role in pathogenesis of AD. Oxidative stress in dementia relates to increased redox active sources such as some transition metals, in particular iron, in the early stages of AD [23]. The most likely mechanism by which amyloid may increase oxidative stress in vitro refers to its ability to bind iron. Thus, it has been suggested that a very large amount of iron could be bound at the neuronal RNA level and numerous studies have indicated an oxidation process of RNA in patients with AD [23]. Given its rapid turnover, neuronal RNA has become one of the most used methods to observe the redox balance status and oxidative stress in the brain. Finally, correlation analysis indicated that these metal abnormalities can be interrelated, participating in common processes such as oxidative stress, altered homeostasis, and uptake into brain, as well as impaired glucose metabolism [29].

Protein levels of hepcidin, the iron-homeostatic peptide, ferroportin, and the iron exporter were significantly reduced in hippocampal lysates from AD brains. In AD brains, hepcidin expression was reduced and restricted to the neuropil, blood vessels, and damaged neurons. In the APP-tg mouse immunoreactivity for ferritin light-chain, the iron storage isoform was initially distributed throughout the brain and as the disease progressed, it was accumulated in the core of amyloid plaques. In human and mouse tissues, extensive AD pathology with amyloid plaques and severe vascular damage with loss of pericytes and endothelial disruption was observed. In AD brains, hepcidin and ferroportin were associated with heme-positive granular deposits in the region of damaged blood vessels [30].

Huperzine A (HupA), a natural inhibitor of acetylcholinesterase derived from a plant, is a licensed anti-AD drug in China. In addition to acting as an acetylcholinesterase inhibitor, HupA possesses neuroprotective properties. Studies showed that the neuroprotective effect of HupA was derived from a novel action on brain iron regulation. HupA treatment reduced insoluble and soluble beta-amyloid levels, ameliorated amyloid plaques formation, and hyperphosphorylated tau in the cortex and hippocampus of APPswe/PS1dE9 transgenic AD mice. Besides, HupA decreased beta-amyloid oligomers and amyloid precursor protein levels and increased A disintegrin and metalloproteinase domain 10 (ADAM10) expression in these treated AD mice [31].

The pathological features of the common neurodegenerative conditions AD and PD are all known to be associated with iron dysregulation in regions of the brain where the specific pathology is most highly expressed [32].

Neuropathology plays a key role in characterizing the pathogenesis of neurodegenerative diseases including forms of neurodegeneration with brain iron accumulation (NBIA). Despite important differences, several genetically diverse forms of NBIA nevertheless share common features in addition to iron deposition, such as the presence of neuroaxonal spheroids. Multiple forms of NBIA also demonstrate tau or *α*-SN pathology, suggesting parallels with both AD and PD. Perls' staining of brain tissue shows a wide spread perivascular deposition of iron and intracellular iron accumulation largely confined to the globus pallidus and substantia nigra, in pantothenate kinase-associated neurodegeneration (PKAN), phospholipase-associated neurodegeneration, mitochondrial membrane protein-associated neurodegeneration, beta propeller protein-associated neurodegeneration, and neuroferritinopathy and aceruloplasminemia. Iron deposition occurs to a lower extent in the substantia nigra. Ferritin staining of both neurons and astrocytes is prominent in PKAN and in general mirrors the pattern seen by Perls' staining. Affected neurons accumulate iron leading to a coarse intracellular appearance that recedes in degenerating cells. Examination of several cases in different stages of disease has indicated that iron deposition begins in the caudate, putamen, thalamus, and dentate nucleus but subsequently spreads throughout the cortex with the frontal and temporal cortices being most profoundly affected [33].

Iron deposition has been found in dopaminergic neurons with *α*-SN [34]. *α*-SN also synthesizes neuromelanin which is associated with iron storage and binds iron-forming stable complexes to sequester large amounts of iron in dopaminergic neurons, thus, resulting in elevated iron levels. Excess iron in dopaminergic neurons can accelerate toxic *α*-SN fibril formation, leading to cellular dysfunction [35]. Ferritin and neuromelanin may contribute to neuroprotection [36], since free cytosolic iron can trigger oxidative stress and promote *α*-SN deposition in Lewy bodies [28, 37].

A recent study in vitro reported that the upregulation of divalent metal transporter 1 without iron-response element (DMT1-IRE) and the increase in DMT1-IRE mediated iron influx play a key role in L-DOPA-induced neurotoxicity in cortical neurons [38]. More importantly, elevated expression of a DMT1 isoform has been found in *α*-SN of PD patients [39], which may be associated with iron accumulation. Similarly, in 1-methyl-4-phenyl-1,2,3,6-tetrahydropyridine-treated mice, a PD model, increased DMT1 expression was found in the ventral mesencephalon, followed by corresponding iron deposition and dopaminergic cell loss. Thus, DMT1 plays an instrumental role in iron accumulation and subsequent oxidative stress-mediated cell damage. Neurons and glial cells appear to export iron via a GPI-anchored form of ceruloplasmin (CP). Quite recently, Jin et al. reported that a decrease in serum CP levels may specifically promote iron deposition in *α*-SN of PD patients [40]. Genetic and pharmacological experiments have proven that chelation of excess iron may be an effective therapy for PD. These findings have led to the development of therapeutic iron chelators for the treatment of NDs.

## 6. Oxidative Stress/Mitochondrial Dysfunction

Excess free radicals can cause neurodegenerative pathological changes of the type through lipid peroxidation reactions. Some evidences showed that free radicals may be involved in the mechanisms of AD and that higher levels of lipid peroxidation products may be in the central nervous system in AD patients [22, 41]. In animal models of AD, increase in lipid peroxidation and elevated levels of reactive oxygen species and decrease in the neurons survival ratio have been shown [42]. Some studies showed that oxidative stress may lead to intralysosomal induction of amyloid to destabilize lysosomal membranes and be indirectly involved in the amyloid genesis, resulting in cell death. These findings have shown a clear link between oxidative stress and pathogenic acroautophagal processes in AD [43].

Accumulation of reactive oxygen species is associated with mitochondria dysfunction and free radicals can be produced by mitochondrial biochemical reactions in AD. The importance of mitochondria may be fundamental in nerve cell survival through the control exercised both on energy metabolism and on various apoptotic pathways. Thus, mitochondria are the most important place producing reactive oxygen species (ROS) in AD [23]. Mitochondria could be considered the central pawn in AD. There were many biochemical changes in the brain of patients with AD, for example, the oxidative stress and mediating intrinsic cellular apoptosis. In this way, morphological analysis showed a clear relationship between the reduction in the number and size of mitochondria and AD [23]. So mitochondrial dysfunctions created serious metabolic disturbances in cellular life that prevent normal functioning of neurons. Mitochondria may initiate cell apoptosis and result in AD [23].

Oxidative stress is also a key theory in PD mechanisms. When a cell is in oxidative stress and the amount of ROS exceeds a certain threshold, the cell does not function effectively which leads to cell death [44]. Studies found some markers of oxidative stress in serum and CSF of PD patients and dopaminergic neurons are particularly susceptible to high level of ROS [44, 45]. Otherwise, crucial antioxidants such as glutathione are low in dopaminergic neurons [46].

Oxidative stress relates to mitochondrial dysfunction; mitochondria are the main producers of ROS in brain [47, 48]. Studies have demonstrated that mitochondrial DNA was altered in some PD cases and that this alteration raised the risk of the disease by increasing ROS formation [49]. Deficiencies of mitochondrial complex I have been noted in brain and other tissues in postmortem PD studies [50]. Particularly, PD animal models from the toxin MPTP create Parkinsonism through interaction with mitochondrial complex I [51]. Thus these findings also show a link between oxidative stress and mitochondrial dysfunction in PD.

## 7. Neuroinflammation

In general, inflammation is a protective response to various cell and tissue injuries. If this response is uncontrolled, the effect initiates excessive cell and tissue damages that result in destruction of normal tissue and chronic inflammation [52]. Alzheimer's disease (AD) and Parkinson's disease (PD) are also the brain inflammatory diseases, which are characterized by “redox state” imbalance and chronic inflammation, a major cause of cell damage and death.

Several studies have shown that brain cells like microglia and astrocytes induce and release diverse inflammatory mediators in response to oxidative stress [53, 54]. An increased number of activated microglial cells have consistently been reported in PD, which may have a deleterious effect on dopaminergic neurons [55]. Most studies have demonstrated that microglial cells play an important role in neuroinflammation and neurodegeneration; accumulating evidence has also demonstrated the characteristic changes of astrocytes in neurodegenerative diseases such as dementia [54, 56]. The senile and neurotic plaque of AD is accompanied by inflammatory responses in activated glial cells. The activated microglia produce several inflammatory mediators including COX-2/prostaglandins (PGs), iNOS/nitric oxide (NO), or cytokines as well as neurotoxic substances, which are thought to be responsible for brain injuries and diseases including AD and neural death due to the exposure of LPS, interferon-*γ*, or *β*-amyloid [55]. Thus, microglia and astrocytes play an important role in PD and AD.

ROS act as a critical signaling molecule to trigger inflammatory responses in central nervous systems (CNS) through the activation of the redox-sensitive transcription factors, including nuclear factor-*κ*B (NF-*κ*B) and activator protein-1 (AP-1) [53, 57]. Excessive production of ROS by mitochondria and NADPH oxidase (Nox) is usually thought to be responsible for tissue injury associated with a range of brain injury, inflammation, and degenerative diseases such as AD [57]. Many of the well-known inflammatory target proteins, including matrix metalloproteinase-9 (MMP-9), cytosolic phospholipase A2 (cPLA2), cyclooxygenase-2 (COX-2), inducible nitric oxide synthase (iNOS), and adhesion molecules, are associated with oxidative stress (ROS generation) induced by proinflammatory factors such as cytokines, peptides, infections, and peroxidants [52, 53, 57].

So neuroinflammation mechanism plays an important role in PD and AD; microglia and astrocytes induce and release diverse inflammatory mediators in response to oxidative stress. ROS act as a critical signaling molecule to trigger inflammatory responses.

## 8. Locus Coeruleus (LC)

Norepinephrine released from LC terminals produces diverse effects based on the adrenoreceptor (AR) on which it acts. The LC is severely affected in neurodegenerative disorders such as AD and PD [58].

In addition to the loss of cholinergic neurons in AD, there is a significant loss of noradrenergic neurons in the LC [59, 60]. The LC innervates many forebrain regions including the cortex and hippocampus, two regions that were severely affected in AD [45]. Some studies indicated that the loss of LC noradrenergic neurons may occur early in the progression of AD [61, 62] before the onset of cognitive impairment. Animal models of familial AD also demonstrated the importance of the LC in the early stages of AD [63].

In PD, postmortem examination also demonstrated a significant loss of noradrenergic neurons in the LC [60, 64]; this loss is equal to or greater than the neuronal loss observed in the dopaminergic *α*-SN region [64]. The loss of LC noradrenergic neuron is also earlier than the loss of dopaminergic neurons in the progression of PD [64, 65]. Although administration of L-DOPA to patients with PD can alleviate motor symptoms, L-DOPA cannot alleviate the nonmotor symptoms associated with LC neuronal loss [66].

So, AD and PD share one major neuropathological iteration, a significant reduction in LC noradrenergic neurons [58, 60, 64]. The reduction of LC noradrenergic neurons occurs early in the progression of both of these disorders [61, 65].

## 9. Nicotinic Receptors

Nicotinic receptors may be associated with AD and PD. Nicotinic acetylcholine receptors (nAChRs) are members of the Cys-loop superfamily of pentameric ligand-gated ion channels, which include GABA (A and C), serotonin, and glycine receptors. Currently, 9 alpha (*α*2–*α*10) and 3 beta (*β*2–*β*4) subunits have been identified in the central nervous system (CNS) and these subunits assemble to form a variety of functional nAChRs. In the CNS, nAChRs play crucial roles in modulating presynaptic, postsynaptic, and extrasynaptic signaling and have been found to be involved in a complex range of CNS disorders including AD and PD. Therefore, the interest of the development of drugs that modulate nAChR functions with optimal benefits and minimal adverse effects is growing [67].

## 10. Conclusion

Although AD and PD have markedly different clinical and pathological features, many mechanisms involved in AD and PD may be the same, such as genes, *α*-synuclein protein, tau protein, oxidative stress, mitochondrial dysfunction, iron, and locus coeruleus. Common mechanisms that shared in AD and PD are supported by many scientific observations through biochemical, genetic, and molecular studies. Thus, there are many reports to prevent or treat these diseases by inhibiting *α*-synuclein protein and tau protein, accumulating and activating antioxidant system, antioxidative stress, specific mutations in specific genes, alterations in mitochondrial disturbances, and so on. AD and PD are neurodegenerative disorders; in future, the hypothesis of more genes or proteins may be involved in AD and PD. Despite some observations of these therapies in AD and PD, treatment studies have thus far failed to prove a clear benefit from the treatment. In conclusion, there is an increasing need for further research regarding inhibiting *α*-synuclein protein and tau protein accumulation, activity of antioxidant system, antioxidative stress, specific mutations in specific genes, alterations in mitochondrial disturbances, and so on.

## Figures and Tables

**Table 1:** Difference between AD and PD.

	Clinical clues	Classic pathological features
AD	Learning and memory difficulties	Amyloid beta-protein
	Frontal executive function	Neurofibrillary tangles
	Language abilities	

PD	Rest tremor	Degeneration of dopamine neurons
	Bradykinesia	Lewy body (LB)
	Rigidity	
	Gait impairment	
